# Efficacy of cemiplimab and lenvatinib combination in metastatic cutaneous squamous cell carcinoma: A case report

**DOI:** 10.1016/j.jdcr.2025.11.021

**Published:** 2025-11-25

**Authors:** Camille Decaestecker, Océane Babin De Lignac, Vivien Hébert, Raphaël Janela-Lapert

**Affiliations:** Department of Dermatology, CHU Rouen, Rouen, France

**Keywords:** combination therapy, complete response, skin cancer

## Introduction

Cutaneous squamous cell carcinoma (cSCC) accounts for approximately 20% of all skin cancers.^1^ Its prevalence and incidence are steadily increasing, primarily due to population aging and sun exposure habits. The risk of locoregional metastases (lymph node or in-transit), and more rarely distant metastases, is estimated at 2% to 5% at 5 years.^2^ First-line treatment of metastatic cSCC is based on anti-PD-1 immunotherapy (pembrolizumab or cemiplimab), with an efficacy rate of approximately 60%.^3^ Other therapeutic options include radiotherapy, chemotherapy (platinum-based agents), and EGFR inhibitors (cetuximab). The therapeutic efficacy of lenvatinib, a tyrosine kinase inhibitor in combination with anti-PD1-agents has been documented across several metastatic malignancies, including endometrial, renal, gastric, and melanoma cancers.^4^, ^5^, ^6^ Notably, the combination of pembrolizumab and lenvatinib had also demonstrated clinical benefit efficacy in head and neck squamous cell carcinoma.^7^ We report the case of metastatic cSCC resistant to conventional therapies that responded to the combination of cemiplimab and lenvatinib.

## Case report

**Fig 1:**
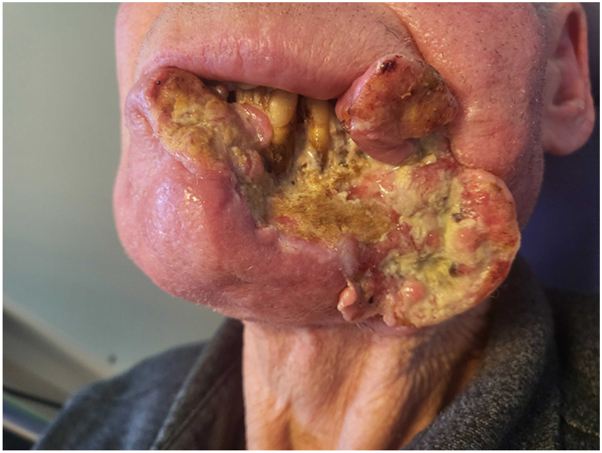
Rapid and destructive local progression after 5 cycles of cemiplimab monotherapy.

**Fig 2:**
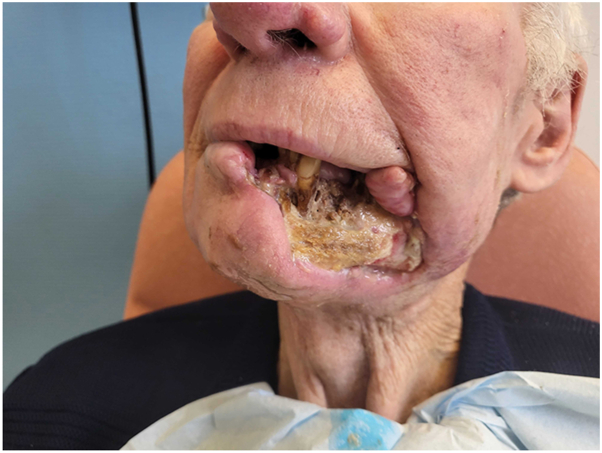
Complete oncologic response with osteoradionecrosis after 5 months of combined cemiplimab and lenvatinib therapy.

**Fig 3:**
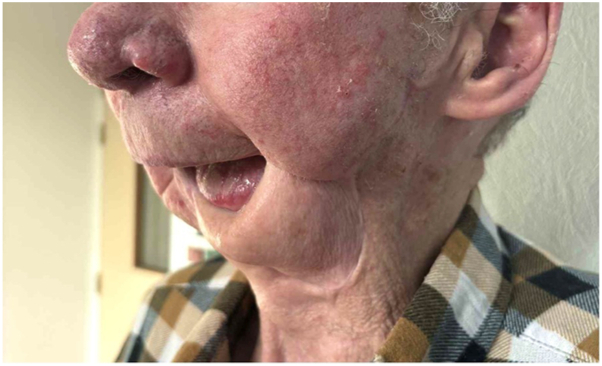
Ongoing complete response on cemiplimab monotherapy, 17 months after lenvatinib discontinuation.

**Fig 4:**
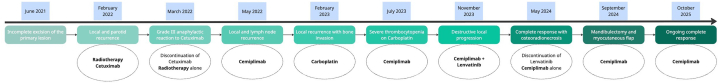
Clinical and therapeutic history.

A patient in their 70s had been followed for 3 years for a cSCC of the lower lip, initially treated surgically with incomplete excision. Local and parotid recurrence developed, treated with radiotherapy and cetuximab. A grade III anaphylactic reaction occurring 60 minutes after initiation of the first cetuximab infusion at a dose of 800 mg, contraindicated further administration of the drug. Ongoing local and retromandibular lymph node progression led to the initiation of cemiplimab at a dose of 350 mg every 3 weeks. After 14 cycles, PET-CT imaging revealed disease progression, with increased hypermetabolic activity involving the skin and subcutaneous tissues of the lip, along with osseous invasion of the ipsilateral horizontal ramus of the mandible. Given the contraindication to further radiotherapy, related to prior irradiation complicate by osteoradionecrosis, and the impossibility of surgical management, monotherapy with carboplatin at a dose of 800 mg every 4 weeks was initiated. Treatment was discontinued after 5 cycles due to poor tolerance, marked by severe thrombocytopenia and cemiplimab was reintroduced. Because of the rapid progression of the initial lesion (Fig 1), lenvatinib 20 mg daily was added to cemiplimab. After 2 months of combination therapy, a complete response was observed, with full remission of the tumor and resolution of lymphadenopathy on PET-CT imaging. The response time supports the efficacy of the cemiplimab and lenvatinib combination in this patient. However, at 5 months, impaired healing with bone exposure prompted several biopsies, which revealed bone necrosis, granulation tissue and ulcerated epithelium, with no evidence of malignancy (Fig 2). Lenvatinib, known to impair wound healing, was discontinued. The osteoradionecrosis was managed with a segmental mandibulectomy and reconstruction of the orofacial defect using a pectoralis major myocutaneous flap. Histopathologic examination of the resected specimen revealed no evidence of recurrent or residual carcinoma (Fig 3). At 17 months following discontinuation, no recurrence was observed (Fig 4).

## Discussion

The efficacy of anti-PD-1 agents in metastatic cSCC has been well established. However, therapeutic options remain limited in cases of treatment failure, often yielding poor long-term outcomes and unfavorable tolerability profiles. Lenvatinib is a tyrosine kinase inhibitor with anti-angiogenic properties, which may account for its potential to impair wound healing.^8^ The rationale for its use in combination therapy for refractory cSCC is based on the marked neovascularization frequently observed in this cancer type, particularly in patients with no remaining standard treatment options. To our knowledge, this is the first reported case of a metastatic cSCC achieving a complete response with the combination of lenvatinib and cemiplimab. This therapeutic approach may represent a promising option in the context of treatment-refractory cSCC. Although, caution is advised due to reported risks of impaired wound healing associated with lenvatinib, particularly in patients with cSCC who frequently undergo radiotherapy.

## Conflicts of interest

None disclosed.
